# Supporting Treatment decision making to Optimise the Prevention of STROKE in Atrial Fibrillation: The STOP STROKE in AF study. Protocol for a cluster randomised controlled trial

**DOI:** 10.1186/1748-5908-7-63

**Published:** 2012-07-06

**Authors:** Melina Gattellari, John M Worthington, Dominic Y Leung, Nicholas Zwar

**Affiliations:** 1 Ingham Institute, Liverpool, Australia; 2 School of Public Health and Community Medicine, The University of New South Wales, Sydney, Australia; 3 South Western Sydney Clinical School, The University of New South Wales, Sydney, Australia; 4 Department of Neurology, Liverpool Health Service, South Western Sydney Local Health Network, Liverpool, Australia; 5 Department of Cardiology, Liverpool Health Service, South Western Sydney Local Health Network, Liverpool, Australia

**Keywords:** Atrial fibrillation, General practice, Anticoagulation, Decisional support

## Abstract

**Background:**

Suboptimal uptake of anticoagulation for stroke prevention in atrial fibrillation has persisted for over 20 years, despite high-level evidence demonstrating its effectiveness in reducing the risk of fatal and disabling stroke.

**Methods:**

The STOP STROKE in AF study is a national, cluster randomised controlled trial designed to improve the uptake of anticoagulation in primary care. General practitioners from around Australia enrolling in this ‘distance education’ program are mailed written educational materials, followed by an academic detailing session delivered via telephone by a medical peer, during which participants discuss patient de-identified cases. General practitioners are then randomised to receive written specialist feedback about the patient de-identified cases either before or after completing a three-month posttest audit. Specialist feedback is designed to provide participants with support and confidence to prescribe anticoagulation. The primary outcome is the proportion of patients with atrial fibrillation receiving oral anticoagulation at the time of the posttest audit.

**Discussion:**

The STOP STROKE in AF study aims to evaluate a feasible intervention via distance education to prevent avoidable stroke due to atrial fibrillation. It provides a systematic test of augmenting academic detailing with expert feedback about patient management.

**Trial registration:**

Australian Clinical Trials Registry Registration Number: ACTRN12611000076976.

## Background

Atrial fibrillation (AF) is the most common heart arrhythmia with a prevalence of around 5% in those aged over 65, increasing to 10% in those older than 75 years [1]. AF confers the highest risk for ischaemic stroke of all traditional risk factors, including congestive heart failure, diabetes, and hypertension [2]. In Australia, at least one in four patients admitted to hospital with an ischaemic stroke has AF [3]. Mortality in stroke patients with AF is around twice the rate in those without it; by 12 months, 40% of ischaemic stroke patients with AF will have died [3]. Moreover, survivors of AF-related stroke spend a significantly longer time in hospital [3].

Meta-analyses of randomised controlled trials of antithrombotic medication demonstrate that, in the average AF patient with a 5% chance of an ischaemic stroke each year, oral anticoagulation with adjusted-dose warfarin reduces this annual risk by 67%, in relative terms, to approximately 1.7% [4]. In contrast, antiplatelet medication, such as aspirin, reduces the risk by only 21%, to approximately 4% [4]. Despite over 20 years of evidence supporting lifelong anticoagulation as the treatment of choice for most patients with AF [4-6], 50%–60% of patients do not receive anticoagulants even if they are at high risk of stroke [7,8]. An Australian study of 218 consecutively admitted patients with AF reported that only 20% were using warfarin on admission [9], although inclusion of an unspecified number of newly diagnosed AF cases would have influenced the reported proportion of under use [9]. In a Canadian stroke unit, 90% of admitted ischaemic stroke patients with AF were not receiving anticoagulation or were insufficiently anticoagulated at the time of their stroke [10]. In one Australian stroke unit, 68% of ischaemic stroke patients with known AF before admission either were not anticoagulated or were sub-optimally anticoagulated at the time of their stroke [11]. This persistent evidence–practice gap contributes to the burden of preventable stroke.

Clinicians are often reluctant to prescribe anticoagulation for AF, particularly if patients are elderly or have a heightened risk of falls or other perceived risk of major bleeding [12-19]. However, many patients considered unsuitable for anticoagulation also have an elevated risk of ischaemic stroke, and the risk-benefit trade-off favours anticoagulation in the majority of patients with AF [20-24].

Few studies have evaluated implementation strategies specifically designed to increase the uptake of oral anticoagulation for AF. McAlister *et al.*[25] reported an increase in antithrombotic prescribing at three months in general practice patients receiving a decision aid. The difference was not sustained at 12 months after randomisation. In a nonrandomised study of Australian primary healthcare physicians in one geographical region of Tasmania, guideline dissemination followed by academic-detailing visits significantly increased prescribing rates of adjusted-dose warfarin compared to a control region [26]. Two other studies in the primary healthcare setting addressed several cardiovascular risk factors, without focusing specifically on AF management [27,28]. Ornstein *et al.*[27] tested the effect of computerised guidelines, reminder systems, and audit and feedback on prescribing behaviour in managing several cardiovascular risk factors. Posttest differences between intervention and control groups were not statistically significant. Another trial found that locally adapted guidelines, an educational seminar delivered by local opinion leaders, educational materials, and an offer of an educational outreach visit did not increase primary healthcare physicians’ adherence to prescribing guidelines for patients with a history of transient ischaemic attack (TIA) or AF [28]. The outcome measure in this study did not distinguish between anticoagulant and antiplatelet prescribing.

Other randomised evaluations of implementation strategies have been carried out with stroke survivors to optimise secondary prevention [29-33]. These have included few AF patients (n = 6 to n = 99) [29-33], and of the two studies reporting outcomes for AF patients, neither found statistically significant improvements in anticoagulant prescribing [32,33].

Specialist decisional support for general practitioners (GPs) merits further investigation. A recent evaluation of a shared-care model for stroke survivors involved liaison between GPs and stroke specialists, facilitated via a nurse specialist. The study found significant improvements in the management of secondary stroke risk and, for AF patients, a nonsignificant improvement in anticoagulation prescribing attributable to the intervention [32]. However, the total number of AF patients in this study was small (n = 32). While the multifaceted nature of the study intervention precludes the identification of effective components, it suggests that linking GPs with stroke specialists could enhance clinical decision making. An observational study also reported a significant effect of collaborative involvement between specialists and GPs on appropriate anticoagulation prescribing, providing further support for the hypothesis that specialist input may be effective in improving the management of AF patients [34].

In another study, known as DESPATCH, we are evaluating the effect of expert decisional support to promote the uptake of anticoagulation using feedback from clinical experts in stroke medicine [35]. However, in the DESPATCH study, decisional support is embedded within a multifaceted intervention including written educational materials, three telephone academic-detailing sessions, and a workshop/seminar. We have designed the STOP STROKE in AF study to systematically assess the impact of expert decisional support on the management of AF in a national study.

## Methods

Please see attached file (Additional file 1) for the study flow diagram.

### GP recruitment

We obtained contact details of GPs from a commercially available database [36], which sources information from professional colleges, medical directories, and state medical boards that register all practising clinicians in Australia. After an initial pilot with 100 randomly selected GPs to determine the likely participation rate, we undertook recruitment in two phases, obtaining lists of 3,000 randomly selected GPs from across Australia in 2010 and 2,501 in 2011 (with those selected in 2010 removed from the sampling frame). GPs who were participating in another implementation trial about AF we were carrying out [37] were excluded from the randomly selected samples (n = 10 in 2010 and n = 13 in 2011).

GPs were mailed a brief introductory letter inviting them to complete a registration form, to be returned by fax or an enclosed business reply paid envelope, advising us of their interest in an education program about stroke prevention. During the first (*i.e.*, in 2010) and second phases of recruitment (*i.e.*, in 2011), we carried out two randomised trials of response-aiding strategies, both of which received ethics approval as amendments to the main study protocol. During the first phase, a statistician independent of the research team randomised 2,250 GPs into three equal-sized groups (n = 750 for each group). The first group was mailed the introductory letter and registration form in an institutional 110 mm by 220 mm-sized envelope (DL-sized) with the university logo and address printed in black and white on the top left-hand corner. The second group was mailed the letter and form in the institutional envelope and also received complimentary copies of resources provided free of charge by the National Stroke Foundation (NSF) of Australia. The third group was also mailed the NSF resources with their letter and form but received these via an Australia Post “Express Post” envelope representing “priority mail”. The remaining 740 GPs were not included in this trial and were mailed the introductory letter and registration form at a different time point to those selected to test response-aiding strategies. During the second wave of recruitment, 2,488 GPs were randomly divided to receive the cover letter and registration form printed on pale yellow (n = 1,247) or white paper (n = 1,240). Letters were mailed using the institutional DL-sized envelopes with logo and address printed in black and white. This trial was separately registered (ACTRN12611000259943).

GPs who returned their registration forms were mailed an information sheet and a consent form, advising that consenting GPs would be invited to participate in an educational session delivered by a medically trained peer via telephone, after which they would be randomised to receive specialist feedback about the de-identified patient cases (see description of intervention below). GPs who returned a signed consent form by fax or enclosed reply paid envelope were then mailed educational materials in advance of a telephone educational session to be delivered by medical peers. Only those GPs who complete the telephone educational session, providing pretest data, are considered recruited and eligible for randomisation. During the initial stages of the first recruitment phase (*i.e.*, in 2010), GPs returning their registration form were telephoned in advance of receiving the information sheet and consent form, and those GPs not returning a consent form within a minimum of two weeks were mailed a second copy. However, due to resource constraints, this follow-up protocol was abandoned.

### Academic detailing

Enrolled GPs receive printed education materials (see Additional file 2) prior to being contacted by a medically trained peer who carried out a standardised telephone educational session covering key issues concerning the management of AF (see Additional file 3). Topics include the epidemiology of AF [1-3], risk stratification using the validated tools [37], and information about the benefits and risks of antithrombotic medications used to manage AF, including adjusted-dose warfarin and antiplatelet medication, such as aspirin and/or clopidogrel [38-44]. During 2011, the information was revised to include information about a revised risk stratification scheme [45] and dabigatran [46-48], a newly available fixed-dose anticoagulant that received regulatory approval in Australia for stroke prophylaxis in AF in May 2011.

GPs were advised in advance of the telephone educational session to identify patients with AF who are over the age of 65 and not receiving anticoagulation or for whom anticoagulation management had presented difficulties. GPs enrolled during the first phase of recruitment (*i.e.*, in 2010) were advised to select around three or four patients for discussion with the medical peer, while those recruited during the second phase (*i.e.*, from 2011 onwards) were advised to select a minimum of five patients.

At the conclusion of the telephone educational session, the medically trained peer proceeded through a standardised patient *pro forma*, collecting de-identified information about patient demographics, stroke risk factors, other comorbidities, current antithrombotic medications, and issues relevant to anticoagulation use. Medically trained peers calculated the patient’s CHADS_2_ score with participating GPs, providing general feedback based on evidence-based guideline recommendations, according to patients’ CHADS_2_ scores [24]. The CHADS_2_ score assigns two points for prior stroke or TIA and one point each for the following risk factors: congestive heart failure, history of hypertension, age over 75, and diabetes. Key safety messages about stroke risk in paroxysmal AF, hypertension, and use of antithrombotic treatment in patients with a history of spontaneous intracranial haemorrhage were also communicated. From late 2011, medical peers also communicated a safety alert issued by the peak Australian drug regulatory body, the Therapeutics Goods Administration (TGA), about the risks of using dabigatran, a newly available oral anticoagulant, in the elderly and in patients with renal impairment [49].

### Pretest data collection

Before commencing the academic-detailing session, medical peers noted GPs’ gender and asked participants to report the number of doctors in their practice and whether they were practising full-time or part-time. Medical peers asked GPs whether they had any of the following resources available to assist them in managing AF patients receiving warfarin: a nurse to monitor and recall patients when needed; automated or computerised reminders for noting when patients need to have their International Normalised Ratios INR levels tested; point-of-care INR testing; pathology service anticoagulation management; or hospital clinical anticoagulation management, practice-based register, or utilisation of a formal Australian government program whereby pharmacists visit patients to monitor compliance with medications. A modified version of the Provider Decision Process Assessment Instrument [50] measuring GP levels of decisional conflict regarding warfarin use was administered. GPs were asked to indicate their level of agreement (agree, disagree, neither) to five statements (‘Whether or not to prescribe warfarin is a difficult decision to make’, ‘For most patients it is clear that warfarin is the best treatment’, ‘It is often difficult to decide if the benefits of warfarin outweigh the risks, or vice versa’, ‘Generally, patients fully appreciate the benefits of warfarin’, ‘Generally, patients fully appreciate the risks of warfarin’). Finally, medical peers asked GPs whether they had participated in formal educational activities about AF in the previous 12 months.

The de-identified patient *pro forma* document contributed to the pretest patient-level data for this study. Information on patient age and sex was first elicited. GPs were then asked to clarify whether the patient had paroxysmal or chronic AF (a response of unsure was permitted) and whether the patient had underlying valvular heart disease or nonvalvular AF. Medical peers asked GPs to indicate if the patient had thyrotoxicosis (yes/no/unsure). Relevant medical history ascertaining stroke risk factors to enable calculation of the CHADS_2_ score was elicited. History of myocardial infarction, peripheral artery disease, aortic plaque, coronary artery disease, and high cholesterol/hyperlipidemia was also noted, as was current smoking status.

Relevant comorbidities reflecting actual or perceived contraindications to anticoagulation were also elicited. These included the following: falls history (without and without injury), a history of multiple falls, anaemia, recent or current upper gastrointestinal (GI) bleeding within the last month, history of upper GI bleeding, history of lower GI bleeding, history of GI bleeding (location not specified), a treated cause of GI bleeding, cerebral haemorrhage, hepatic insufficiency/moderate to severe liver disease, impaired kidney function, chronic dialysis, renal implantation, abnormal serum creatinine (>200 ųmol/L), hepatic derangement on biochemical testing, excessive alcohol intake (defined as eight or more units per week), coagulopathy, thrombocytopaenia, dementia or cognitive impairment (with and without supervised care), insertion of coronary artery stent (drug-eluting, bare metal, or stent of unknown type), and nonadherence with medication or management. Comorbidities assessing bleeding risk as described above were derived from a standardised scheme [51]. GP participants could nominate other comorbidities they considered relevant for specialist feedback. The *pro forma* elicited information on current antithrombotic medications; previous use of anticoagulation with adjusted-dose warfarin; and, where relevant, reasons for not using warfarin, for ceasing warfarin, and adverse events whilst receiving warfarin (minor bleeding not requiring hospitalisation, major blood loss requiring hospitalisation, intracranial or intracerebral haemorrhage, including subarachnoid haemorrhage; ischaemic stroke; TIA or amaurosis fugax; sub-therapeutic INR levels; supra-therapeutic INR levels; consistently unstable INR levels). Rate- or rhythm-control medications, non-steroidal anti-inflammatory drugs (NSAIDs) including COX-2 inhibitors, paracetamol, alternative medications, and other over-the-counter medications in current use were also noted. From 2011 onwards, GPs were asked to note, where relevant, adverse events whilst receiving anticoagulants other than warfarin (most likely dabigatran).

### Randomisation

A statistician external to the study group is carrying out randomisation after participants complete academic-detailing sessions. Randomisation is being stratified by the number of cases GPs identify (≤2 or >3). Results of randomisation are communicated to the study coordinator (MG) who assigns GPs to their respective groups. The statistician involved in the analysis of the trial will receive the randomisation schedule directly from the statistician carrying out randomisation. Block randomisation is being used to control for date of entry into the trial. Block size will be disclosed to the study team only during the write-up of results.

### Expert decisional support

Medically trained peers reminded GPs that they will be randomised to receive specialist feedback about patient de-identified cases either prior to or after posttest evaluation. They were advised to consider this component of the intervention as an educational exercise and, therefore, should not have expectations of timely feedback and should seek any urgently required advice through their usual practices. Data from the de-identified *pro forma* documents were mailed to MG, who entered the data and summarised information into a one- to two-page document that was forwarded to experts in either neurology, cardiology and stroke. The experts provided written information that was then mailed to GPs randomised to receive expert decisional support prior to the posttest phase of the study (see Additional file 4 for a hypothetical example). Our model for delivering this aspect of the intervention was informed by an intervention developed by McAlister *et al.*, 2006 [52].

### Waiting list control

GPs randomised to the waiting list control group were mailed a summary of patient de-identified information, identical to the summary received by GPs randomised to the intervention arm. These GPs also received a statement summarising patient ischaemic stroke risk based on the patient’s CHADS_2_ score. Therefore, patient summaries were identical between groups, with the exception of specialist written feedback.

### Posttest data collection

Posttest data collection is scheduled for approximately 12 weeks after GPs have received either the expert decisional feedback or, for those randomised to the waiting list control group, the patient de-identified summary document. GPs receive another copy of the patient de-identified summary sheet (without expert feedback) attached to a brief posttest questionnaire. For each patient, GPs are asked to indicate current antithrombotic treatment from a list of available choices (warfarin, aspirin, clopidogrel, aspirin and clopidogrel, dipyridamole, dipyridamole with aspirin, dabigatran, other antithrombotic for GP to specify, or no antithrombotic) and, if currently receiving warfarin, to report the last six INR test results and corresponding dates of blood collection. In response to a checklist of items, GPs are asked to specify reasons for any given patient not receiving anticoagulation (patient refusal/reluctance, history of traumatic brain haemorrhage, history of spontaneous brain haemorrhage, history of lower GI bleed, history of upper GI bleed, falls history without injury, falls history with injury, history of GI location not specified, age, cognitive impairment, inadequate supervision at home, patient nonadherence with medication, patient nonadherence with monitoring, specialist doesn’t recommend anticoagulation, patient at low risk of stroke, awaiting government subsidisation of fixed-dose anticoagulation with dabigatran, or other reason which GPs are asked to specify).

Whether or not patients are receiving anticoagulation, GPs complete an adapted version of the Provider Decision Process Assessment Instrument [50], consisting of six items assessing GP levels of decisional conflict regarding anticoagulation with adjusted-dose warfarin, with an additional question asking GPs to indicate their level of satisfaction with the current treatment. From 2011 onwards, the posttest evaluation included an additional six questions assessing levels of decisional conflict for a newly available fixed-dose anticoagulant, dabigatran. Posttest data will be entered by personnel blinded to group allocation.

### Primary outcome

The primary outcome is defined as the proportion of patients with AF who, at posttest, are reported by GPs to be receiving anticoagulation.

### Secondary endpoints

#### Appropriate antithrombotic treatment according to stroke risk

In accordance with evidence-based guidelines [38-40], we will use baseline CHADS_2_ scores in those patients aged over 65 years to judge appropriateness of antithrombotic management. Specifically, patients over 65 years of age with a baseline CHADS_2_ score of 0 will be considered to be managed appropriately if receiving aspirin, while those with a baseline CHADS_2_ score of 2 or more will be considered to be receiving appropriate antithrombotic treatment if receiving anticoagulation (either with warfarin or dabigatran). Either anticoagulation or aspirin will be considered appropriate in those with a baseline CHADS_2_ score of 1. The expected few cases of patients younger than 65 years of age will be excluded from this analysis. However, a sensitivity analysis will be carried out applying the CHADS_2_ score to patients younger than 65 years of age, as above, in recognition of inclusion criteria used in recent trials of antithrombotic treatments that included patients younger than 65 where AF was associated with CHADS_2_ stroke risk factors [4-6].

#### Appropriate antithrombotic treatment according to stroke risk incorporating quality-control criteria

In those patients receiving anticoagulation with warfarin, we will only consider anticoagulation to be appropriate for this outcome if GPs, when reporting the six most recent INR results, report an INR result at least monthly and where at least four of the six test results (or two-thirds of results in cases where fewer than six are reported) are within therapeutic range (*i.e.*, within 2.0–3.0). These criteria for appropriate anticoagulation are based on local guidelines [44] and a consistently achieved standard of ‘time in therapeutic range’ reported in clinical trials [4-6], including one trial of general practice patients with an average age of 81 years [6].

#### Further refinement of outcome definition

Combinations of antiplatelet therapies with or without anticoagulation may diverge from standard guideline recommendations but may nonetheless be appropriate in the context of aspirin intolerance, patients with a history of coronary stenting, or in those affected by acute coronary syndromes [42,43,53-55]. We will accept other antiplatelet treatments, such as clopidogrel, dypridamole, and combinations of these treatments, where aspirin would have been considered the evidence-based treatment. Where anticoagulation is considered the evidence-based choice, we will not distinguish between cases where anticoagulation is used alone or in combination with other antithrombotics.

#### Decisional conflict

Scores comprising the seven-item decisional conflict scale, based on a previously validated measure [50] adapted for use in our earlier work and in other studies [13,14], will be summed to produce a total score, whereby higher scores indicate higher levels of decisional conflict (range = 7 to 35). At the time of writing, warfarin remains the only anticoagulant treatment that is available to all Australian residents via Australia’s universal health insurance scheme ensuring government subsidisation of medication. The fixed-dose anticoagulant dabigatran has received regulatory approval for AF but is not currently subsidised by the Australian government. Consequently, the uptake of dabigatran is not expected to be high, and we will restrict this outcome to items assessing decisional conflict about warfarin only, unless uptake of dabigatran is found to be common.

#### Sensitivity analyses

In cases that are lost to follow-up (for example, where GPs do not return their posttest audit questionnaires), we will reanalyse our primary and secondary outcomes relating to prescribing behaviour, firstly, assuming no change in antithrombotic management from baseline to posttest and secondly, assuming the desired outcome was not achieved.

#### Subgroup analyses

We will carry out subgroup analyses for our primary and secondary outcomes according to the following variables: (a) baseline CHADS_2_ scores (0, 1, or ≥2); (b) patient age (<65 years, 65–74 years, 75–84 years, ≥85 years); (c) baseline anticoagulation use (yes versus no); (d) patient sex.

#### Pretest comparisons between groups

We will compare groups on the numbers of patients selected by GPs, patient sex, age (mean and median differences and on categorical groupings <65 years, 65–74, 75–84, ≥85 years), CHADS_2_ scores (0, 1, ≥2, and mean scores), and use of oral anticoagulation (current at pretest, previous use, or never used).

#### Sample size estimate

Our study was powered to detect a 15% difference in anticoagulation rates between groups. We assumed a baseline use of anticoagulation of 50% to produce anticonservative (*i.e.*, larger) estimates of the required sample size. In the absence of a design effect, whereby the sample size would be adjusted for clustering of patients by GP, we required GPs to identify 170 patients per group or 340 in total [56]. As patient cases were clustered by GP, we adjusted our sample size estimate by the design effect *D*_eff_ = 1 + (*m* − 1)ρ [57], where *m* equals the average cluster size and ρ equals the intraclass correlation coefficient. We have selected a value of *m* to produce anticonservative (*i.e.*, larger) estimates of the required GP sample, as the number of GPs needed will have a greater effect on study resources and its feasibility than the numbers of patient cases identified by GPs. Smaller values of *m* will produce a sample size estimate requiring greater numbers of GPs. Assuming an average cluster size of three and selecting an intraclass correlation coefficient of 0.029, informed by results from a previous study [27], our anticipated design effect was 1.06 (*i.e.*, 1 + (3 – 1) × 0.029), producing a required total sample size of 1.06 × 340 = 361. Assuming 20% of posttest audit questionnaires are not returned by GPs, we require data on 452 de-identified patient cases or 226 per group. If GPs each select three patient cases on average, then we will need to recruit 76 GPs per group (*i.e.*, 226/3), or 152 in total.

#### Statistical analysis

Outcomes will be analysed according to the intention-to-treat principle, whereby patients are analysed according to the arm to which their GP cluster was allocated. Clustering will be accounted for at the practice level [57]. A *p* value of < .05 will be used to determine statistical significance of results. Analyses will be carried out by a statistician blinded to group allocation.

#### Ethics approval

The Human Research Ethics Committee of the University of New South Wales has approved the study (UNSW HREC Reference Number 07067).

## Discussion

Long-term anticoagulation for AF remains the treatment of choice to prevent stroke in most people with AF. The STOP STROKE in AF study has an active ‘control’. Information is delivered via academic detailing with tailored feedback about risk stratification and guideline recommendations. The addition of expert decisional support in our intervention arms allows for a direct test of this component on GP self-reported patient management. The national geographical reach of the study and feasible delivery of the intervention, via telephone and mail, are innovations of the study.

Until recently, warfarin has been the only oral anticoagulant available for managing AF. Two fixed-dose anticoagulants, dabigatran [46,47] and rivoroxaban [58], have received approval for stroke prophylaxis in AF by the US Federal Drug Administration (FDA). Apixaban [59], another fixed-dose anticoagulant, is due to be reviewed by the FDA in 2012. It is unclear whether the advent of new drugs will improve anticoagulation rates. In practice, uptake of dabigatran, the first FDA-approved fixed dose oral anticoagulant, has been described as ‘disappointing’, with an estimated 10% of AF patients using the drug in the United States [60]. The cost of the drug, the need for twice-daily dosing, a short shelf-life, the lack of an antidote to reverse acute bleeding, the inability to determine the anticoagulation effects of dabigatran, and the increased risk of GI bleeding, dyspepsia, discontinuation, and possibly myocardial infarction, when compared with warfarin, may limit its broader uptake [58]. Recent warnings from regulators in Australia [48] and the United States [61] have emphasised safety concerns about dabigatran, particularly in those with renal impairment and in elderly patients aged over 75 years. In Australia, AF became an approved indication for dabigatran by the local government regulator, the TGA, after which the company manufacturing the drug offered doctors free medication for a limited period of time, or until the drug became subsidised by the Australian government (whichever occurred first). The Australian government has thus far declined the company’s application to subsidise the drug. Federal agencies in Australia are currently undertaking a further review of the costs and benefits of the new drug and defining the circumstances in which warfarin may be more cost effective. Other fixed-dose anticoagulants that may come to market in the near future, specifically rivaroxaban and apixaban, share some drawbacks of dabigatran; all three lack an antidote to reverse acute bleeding, and apixaban also requires twice-daily dosing.

Prescribing behaviour seems difficult to shift. The South London secondary prevention programme, also called Stop Stroke, enrolled 523 stroke survivors, 99 of whom had AF. Patients were randomised to receive ‘keeping well plans’ summarising risk factors and management strategies, including evidence-based prescribing for managing blood pressure, diabetes, and AF, that were updated over a 12-month period [33]. The intervention had no effect on any of the study outcomes, including anticoagulant prescribing for AF patients.

Clinician fears about the side effects of anticoagulation appear to have greater influence on anticoagulant prescribing than do concerns about stroke risk. Clinicians are less likely to prescribe anticoagulation in patients with AF if one of their AF patients receiving anticoagulation is hospitalised for a major bleed, yet prescribing behaviour is not influenced if untreated AF patients are hospitalised for an ischaemic stroke [62]. In our national Australian survey [13,14], GPs with prior experiences of a haemorrhagic stroke in AF patients report a heightened sense of responsibility for that outcome. In contrast, GPs with untreated AF patients who had a stroke were no more likely to feel responsible for this outcome than other GPs. Further, our survey showed that GPs appeared overly cautious in prescribing anticoagulation in the presence of any perceived risk of bleeding, even where treatment benefits clearly outweighed the risk of harm. Other Australian surveys show that GPs are reluctant to prescribe anticoagulation for nonvalvular atrial fibrillation in the elderly or in the presence of perceived bleeding risks, which would not contraindicate anticoagulation according to available evidence [15-17].

The STOP STROKE in AF study aims to redress clinician wariness and concerns over the use of anticoagulation and support effective decision making to build practitioner confidence and increase the appropriate uptake of anticoagulation in AF.

In addition to our own research, we are aware of one other Australian study evaluating a risk assessment tool in general practice for improving the management of AF [63,64]. It is hoped that these studies will collectively identify effective implementation strategies to better inform GPs on AF management, closing the evidence–practice gap in Australia and elsewhere.

### Progress

Required numbers of GPs have been recruited. We expect posttest data collection to be completed in 2012. Results are expected in 2013.

## Competing interests

The authors state they have no competing interests to declare.

## Authors’ contributions

JMW initiated the group’s interest in developing an intervention in general practice to reduce stroke risk in patients with atrial fibrillation. MG is the lead investigator on the study. MG and JMW had major input into study design and conceptualisation and cowrote the protocol for publication. NZ and DYL provided input into study protocol at the time of its conceptualisation. JMW and DYL led the expert decisional-support component of the intervention and informed outcome assessment. JMW prepared authored information resources. All authors reviewed and provided feedback on the protocol. All authors have read and approved the final submission.

## Supplementary Material

Additional file 1: **Consort flow diagram.** (DOC 30 kb) Click here for file

Additional file 2: **Summary of written information mailed to all GPs.** (DOCX 15 kb) Click here for file

Additional file 3: **Content summary of academic detailing session.** (DOCX 18 kb) Click here for file

Additional file 4: **Example of patient summary and expert feedback.** (DOC 41 kb) Click here for file
