# Supplementary material for: Supporting Treatment decision making to Optimise the Prevention of STROKE in Atrial Fibrillation: The STOP STROKE in AF study. Protocol for a cluster randomised controlled trial
Source: Implement Sci. 2012 Jul 6;7:63. doi: 10.1186/1748-5908-7-63 (PMC3443055; doi:10.1186/1748-5908-7-63)
Supplement: Additional file 1: — Consort flow diagram. (DOC 30 kb) [file 1748-5908-7-63-S1.doc]

**CONSORT Flow Diagram**

**Allocation**

**Posttest**

**Enrollment:**

**2010-2012**

N=5601 GPs approached via mail to take part

Posttest self-audit questionnaires mailed approximately three months after receiving patient summary and feedback document

*Allocated to Expert Decisional Control (n=76 GPs identifying 226 de-identified patient cases)*

-Participation in one academic detailing session via phone

-Mailed patient summary with risk score calculation + expert feedback regarding patient de-identified cases

Posttest self-audit mailed approximately three months after receiving patient summary document

*Required No. of participants*

(n=152 GPs identifying 452 patient de-identified cases) All GPs mailed educational resources

*Allocated to Academic detailing (n=76 GPs identifying 226 de-identified patient cases)*

-Participation in one academic detailing session via phone

-Mailed patient summary with risk score calculation without expert feedback

-Expert feedback mailed after follow-up

**(adapted from** [**http://www.consort-statement.org/consort-statement/flow-diagram0/**](http://www.consort-statement.org/consort-statement/flow-diagram0/) **Last accessed January 2, 2012)**

*Exclusion criteria*

-On extended leave

-In speciality practice (eg travel medicine, sports medicine)

-Participation in another trial [34]

-Not returning consent form
