# Supplementary material for: Supporting Treatment decision making to Optimise the Prevention of STROKE in Atrial Fibrillation: The STOP STROKE in AF study. Protocol for a cluster randomised controlled trial
Source: Implement Sci. 2012 Jul 6;7:63. doi: 10.1186/1748-5908-7-63 (PMC3443055; doi:10.1186/1748-5908-7-63)
Supplement: Additional file 2: — Summary of written information mailed to all GPs. (DOCX 15 kb) [file 1748-5908-7-63-S2.docx]

**Additional File 2: Summary of written information mailed to all GPs**

| **Handout** | **Summary of Content** |
| --- | --- |
| Primary and Secondary Stroke Prevention in Non-Valvular AF (developed by MG and JMW) | - The prevalence of atrial fibrillation - Stroke risk and atrial fibrillation (the CHADS_2_ score) (also prepared as a separate handout) - Evidence-based guidelines and the management of atrial fibrillation - Antithrombotic treatment for atrial fibrillation and the risk of bleeding - Can anticoagulation be safely used in the elderly? Results from the BAFTA study - Antithrombotic treatment for atrial fibrillation and the risk of bleeding - The BAFTA study–main findings - How important is falls risk when prescribing warfarin? - Upper GIT bleeding - Recurrent nosebleeds - What are the contraindications to warfarin use? (also prepared as a separate handout) - Fixed-dose anticoagulation for atrial fibrillation |
| Handout 2: Warfarin in Practice (developed by JMW) | - Drug and food interactions with warfarin (also prepared as a separate handout) - Summary of evidence-based guidelines [37-39] |
| Handout 3: Clopidogrel fact sheet (developed by JMW and MG) | - Summaries of ACTIVE-W [41] and ACTIVE-A studies [42] |
| Handout 4: Warfarin reversal guidelines | - See refs [40] |
