# Supplementary material for: Supporting Treatment decision making to Optimise the Prevention of STROKE in Atrial Fibrillation: The STOP STROKE in AF study. Protocol for a cluster randomised controlled trial
Source: Implement Sci. 2012 Jul 6;7:63. doi: 10.1186/1748-5908-7-63 (PMC3443055; doi:10.1186/1748-5908-7-63)
Supplement: Additional file 3: — Content summary of academic detailing session. (DOCX 18 kb) [file 1748-5908-7-63-S3.docx]

**Additional File 3: Content summary of academic detailing session**

**2010**

**Prompt:** Epidemiology of Atrial fibrillation in ischaemic stroke patients

**Prompt:** Information about the CHADS_2_ score

Guideline recommendations based on the CHADS_2_ score*

Ischaemic stroke risk for each CHADS_2_ stroke score*

Absolute risk reduction with anticoagulation using adjusted-dose warfarin and aspirin when compared with no treatment

**Prompt:** GPs asked two questions about a hypothetical group of patients

over the age of 65 with atrial fibrillation :

1. Characteristics of patients who do not receive warfarin
2. Discussion of evidence regarding perceived barriers (age, falls risk, bleeding risk, cognitive impairment and stroke risk thought to be too low)* *(Note: all topics covered irrespective of GP responses)*
3. Alternative treatments in those not receiving anticoagulation
4. Discussion of evidence about alternative treatments (Aspirin, low, fixed-dose warfarin, dipyridamole+aspirin, clopidogrel+aspirin, dabigatran).* *(Note: all topics covered irrespective of GP responses)*

**Prompt:** Completion of patient proformas

**From 2011 onwards**

**Prompt:** Epidemiology of Atrial fibrillation in ischaemic stroke patients

**Prompt:** Information about the CHADS_2_ score

Guideline recommendations based on the CHADS_2_ score

Ischaemic stroke risk for each CHADS_2_ stroke score

Absolute risk reduction with anticoagulation using adjusted-dose warfarin and aspirin when compared with no treatment

**Prompt:** The CHA_2_DS_2_-VASc score [refs]

**Prompt:** Discussion of BAFTA study* [6]

**Prompt:** Warfarin versus aspirin and bleeding risk*

**Prompt:** GPs asked two questions about a hypothetical group of patients

over the age of 65 with atrial fibrillation :

1. Characteristics of patients who do not receive warfarin
2. Discussion of evidence regarding perceived barriers (eg age, falls risk, dementia) *(Note: topics covered tailored to GP responses)*
3. Alternative treatments in those not receiving anticoagulation
4. Discussion of evidence about alternative treatments. *(Note: Topics covered tailored to GP responses)*

**Prompt:** Completion of patient proformas

*Information regarding BAFTA study and bleeding risk were covered at both time points.
