# Supplementary material for: Supporting Treatment decision making to Optimise the Prevention of STROKE in Atrial Fibrillation: The STOP STROKE in AF study. Protocol for a cluster randomised controlled trial
Source: Implement Sci. 2012 Jul 6;7:63. doi: 10.1186/1748-5908-7-63 (PMC3443055; doi:10.1186/1748-5908-7-63)
Supplement: Additional file 4: — Example of patient summary and expert feedback. (DOC 41 kb) [file 1748-5908-7-63-S4.doc]

**70 year old [female/male] patient with atrial fibrillation [non-valvular] without thyrotoxicosis**

SUMMARY OF PATIENT HISTORY

| **Type of AF** | Paroxysmal |
| --- | --- |
| **Ischaemic stroke risk factors** | Hypertension (controlled)  Diabetes |
| **CHADS_2_ Score** | 2 |
| **Annual Ischaemic Stroke Risk** | 4.0% |
| **Other relevant co-morbidities** | Nil reported |
| **Antithrombotic medications** | Warfarin |
| **Other medications** | Verapamil |
| **Anticoagulant use –Current and past** | Yes |
| **Adverse events/concerns**  **whilst on warfarin** | no major or minor bleeding and no ischaemic stroke events |
| **Reasons for not**  **prescribing warfarin** | NA |
| **Home Medicines Review (HMR) considered?** | No |
| **Webster packaging considered?** | No |
| **GP questions re management/comments** | What is the role of aspirin in this patient, either used as a single antithrombotic or in combination with warfarin? |

**Specialist comments:** ***Annual Ischaemic Stroke Risk: 4.0%***

The risk of stroke is similar for both paroxysmal and chronic AF. Hence, a patient with a CHADS_2_ score of 2 should be on warfrain if they are in sinus rhythm but have a history of paroxysmal AF. Aspirin reduces the relative risk of a stroke in AF by 19% over placebo and hence does have a role – it is better than no antithromotic therapy. However, warfarin has a relative risk reduction over aspirin of ~ 39% and is more effective than aspirin in patients with paroxysmal or chronic AF and a CHADS2 score of 2 or higher. There is no evidence of any additional benefit of combination aspirin and warfarin versus warfarin alone for AF. I would also have concerns about an additive increased risk of bleeding with such a combination.

**Note: While this information is based on a real patient, demographic and disease characteristics have been modified to derive this hypothetical patient case.**
